# Three-dimensional and single-cell sequencing of liver cancer reveals comprehensive host-virus interactions in HBV infection

**DOI:** 10.3389/fimmu.2023.1161522

**Published:** 2023-03-31

**Authors:** Mengbiao Guo, Zhicheng Yao, Chen Jiang, Zhou Songyang, Lian Gan, Yuanyan Xiong

**Affiliations:** ^1^ Key Laboratory of Gene Engineering of the Ministry of Education, Institute of Healthy Aging Research, School of Life Sciences, Sun Yat-sen University, Guangzhou, China; ^2^ Department of General Surgery, The Third Affiliated Hospital, Sun Yat-sen University, Guangzhou, China; ^3^ Nansha-South China Agricultural University Fishery Research Institute, Guangzhou, China; ^4^ College of Marine Sciences, South China Agricultural University, Guangzhou, China

**Keywords:** HBV, liver cancer, host-virus interaction, Hi-C, TAD and A/B compartments, structural variation (SV), transposable element (TE), single cell sequencing

## Abstract

**Backgrounds:**

Hepatitis B virus (HBV) infection is a major risk factor for chronic liver diseases and liver cancer (mainly hepatocellular carcinoma, HCC), while the underlying mechanisms and host-virus interactions are still largely elusive.

**Methods:**

We applied HiC sequencing to HepG2 (HBV-) and HepG2-2.2.15 (HBV+) cell lines and combined them with public HCC single-cell RNA-seq data, HCC bulk RNA-seq data, and both genomic and epigenomic ChIP-seq data to reveal potential disease mechanisms of HBV infection and host-virus interactions reflected by 3D genome organization.

**Results:**

We found that HBV enhanced overall proximal chromatin interactions (CIs) of liver cells and primarily affected regional CIs on chromosomes 13, 14, 17, and 22. Interestingly, HBV altered the boundaries of many topologically associating domains (TADs), and genes nearby these boundaries showed functional enrichment in cell adhesion which may promote cancer metastasis. Moreover, A/B compartment analysis revealed dramatic changes on chromosomes 9, 13 and 21, with more B compartments (inactive or closed) shifting to A compartments (active or open). The A-to-B regions (closing) harbored enhancers enriched in the regulation of inflammatory responses, whereas B-to-A regions (opening) were enriched for transposable elements (TE). Furthermore, we identified large HBV-induced structural variations (SVs) that disrupted tumor suppressors, *NLGN4Y* and *PROS1*. Finally, we examined differentially expressed genes and TEs in single hepatocytes with or without HBV infection, by using single-cell RNA-seq data. Consistent with our HiC sequencing findings, two upregulated genes that promote HBV replication, *HNF4A* and *NR5A2*, were located in regions with HBV-enhanced CIs, and five TEs were located in HBV-activated regions. Therefore, HBV may promote liver diseases by affecting the human 3D genome structure.

**Conclusion:**

Our work promotes mechanistic understanding of HBV infection and host-virus interactions related to liver diseases that affect billions of people worldwide. Our findings may also have implications for novel immunotherapeutic strategies targeting HBV infection.

## Introduction

Hepatocellular carcinoma (HCC) is the most common primary liver cancer and is the third deadliest cancer worldwide (1, 2). Most HCC cases are associated with chronic infection of hepatitis B virus (HBV) that infected >250 million people (2, 3) in Asia. HBV may cause liver cancer by various mechanisms through host-virus interactions (4). HBV DNA can integrate itself into the human genome non-randomly and up-regulate oncogenic driver genes (5–7), such as *TERT*, *KMT2B*, and *CCNE1*. HBV integration is one of the main reasons that HBV infection is difficult to cure.

Various types of viruses, including HBV (8–11), Epstein–Barr virus (EBV) (12), human papillomavirus (HPV) (13), human herpesviruses (HHV) (14), influenza A virus (IAV) (15), human leukemia virus (HTLV-1) (16), and parvoviruses (17), have been reported to have interactions with the genome 3D organization [as reviewed in (18)]. The genome 3D organization coordinately regulated multiple genes expression by 3D chromatin structures, topologically associating domains (TADs, Mbp-scale genomic regions) and A/B compartments (composed of groups of TADs) (19, 20). TADs are insulated regions composed of multiple genes and regulatory elements, such as enhancers and silencers. TAD boundaries are guarded by specialized protein complexes including CCCTC-binding factor (CTCF) and restrict chromatin looping of, for example, enhancers within TADs to prevent them from aberrantly regulate genes outside TADs. Disrupting TADs or their boundaries can cause cancer (21).

In primary hepatocytes, previous research found that short-term (several days) HBV infection cannot change the 3D genome structure (8). However, another recent report in liver cancer cells showed that integrated HBV DNA can form chromatin loop with host genomic DNA (9), which we think may potentially function as oncogenic circular extrachromosomal DNA (ecDNA) that promotes accessible chromatin (22). We thus hypothesize that long-term HBV-related host-virus interactions (especially with HBV integration) can possibly change the human genome 3D organization, especially in liver cancer. However, the global impact of HBV integration on the human genome TADs and the chromatin status (A/B compartments) remains unknown.

Here, we comprehensively investigated HBV infection and host-virus interactions using cell lines and patient tumors of liver cancer, by combining in-house 3D genome sequencing [HiC (20)] in HepG2 (HBV-) and HepG2-2.2.15 (HBV+) cells and public single-cell RNA sequencing (scRNA-seq), together with other large multi-omics datasets. Our work demonstrated that HBV can change the human genome organization globally, related to inflammatory responses, and potentially activated certain host genes and transposable elements (TE, long genomic repeats) (23) specifically to promote viral replication. HBV may both activate oncogenes by reprogramming TADs or A/B compartments of chromatin interactions (CIs) and disrupting tumor suppressors by generating structural variations (SVs).

## Materials and methods

### Data collection

Our Hi-C sequencing reads was deposited in Sequence Read Archive [SRA, accession PRJNA768726 (https://www.ncbi.nlm.nih.gov/bioproject/PRJNA768726)]. TCGA-LIHC RNA-seq data were downloaded from https://portal.gdc.cancer.gov. The scRNA-seq data (seven HCC tumor samples) were downloaded from Genome Sequence Archive with accession CRA002308 (24). ENCODE data were downloaded from https://encodeproject.org. Roadmap Epigenomics data were downloaded from http://www.roadmapepigenomics.org. HepG2 enhancer data were obtained from EnhancerDB (25) and FANTOM5 (26).

### Cell culture

HepG2, HepG2-2.2.15 were obtained from the Cell Bank Type Culture Collection of the Chinese Academy of Sciences (Shanghai, China). All cell lines were maintained in Dulbecco’s modified Eagle’s medium (Gibco, Thermo Fisher Scientific, MA, USA) supplemented with 10% fetal bovine serum (Gibco, Thermo Fisher Scientific, MA, USA) and penicillin-streptomycin antibiotics (Gibco, Thermo Fisher Scientific, MA, USA), and were incubated at 37°C with 5% CO2.

### Hi-C sequencing

Hi-C experiments and sequencing were performed by AnnoRoad (http://en.annoroad.com) following manufacturer’s instructions. Genomic DNA from about 5x10^7^ cells were used for Hi-C library preparation. For crosslinking of cells, we followed these steps: (1) Aspirate the medium and add 22.5 ml of fresh medium without serum per plate. (2) Crosslink the cells by adding 1.25 ml of 37% formaldehyde to obtain 2% final concentration. Mix gently, immediately after addition of formaldehyde. (3) Incubate at room temperature (RT) for exactly 10 min. Gently rock the plates every 2 min. (4) To quench the crosslinking reaction, add 2.5 ml of 2.5 M glycine, mix well. (5) Incubate for 5 min at RT and then incubate on ice for at least 15 min to stop crosslinking completely. (6) Scrape the cells from the plates with a cell scraper and transfer to a tube. (7) Mix cells well and then split the crosslinked cell suspension into aliquots of 25 x 10^6^ cells. (8) Centrifuge the crosslinked cells at 800xg for 10 min. (9) Discard the supernatant by aspiration.

The fixed cells were resuspended in 1 ml of lysis buffer (10 Mm Tris-HCl pH 8.0, 10 mM NaCl, 0.2% Igepal CA-630, 1/10 vol. of proteinase inhibitor cocktail (Sigma)), and then incubated on ice for 20 minutes. Nuclei were pelleted by centrifugation at 4°C, 600x g for 5 minutes, and then washed with 1 ml of the lysis buffer, followed by another centrifugation under similar conditions. After washing twice with restriction enzyme buffer, the nuclei were resuspended in 400 μl of restriction enzyme buffer and transferred to a safe-lock tube. Next, The chromatin is solubilized with dilute SDS and incubation at 65°C for 10 min. After quenching the SDS by Triton X-100 overnight digestion was applied with 4 cutter restriction enzyme (400 units MboI) at 37°C on rocking platform. Next, mark the DNA ends with biotin-14-dCTP and perform blunt-end ligation of crosslinked fragments. The proximal chromatin DNA was religated by ligation enzyme. The nuclear complexes were reversed crosslinked by incubating with proteinase K at 65°C. DNA was purified by phenol-chloroform extraction. Biotin-C was removed from non-ligated fragment ends using T4 DNA polymerase. Fragments was sheared to a size of 200-600bp by sonication. The fragment ends were repaired by the mixture of T4 DNA polymerase, T4 polynucleotide kinase and Klenow DNA polymerase. Biotin labeled Hi-C samples were specifically enriched using streptavidin C1 magnetic beads. The fragment ends were adding A-tailing by Klenow(exo-) and then adding Illumina paired-end sequencing adapter by ligation mix. The Hi-C libraries were amplified by 12-14 cycles PCR and sequenced by the Illumina HiSeq platform with 2×150-bp reads.

### Analysis of TADs, A/B compartments, and SVs using Hi-C sequencing data

BWA (27) was used to map Hi-C sequencing reads to the human reference genome (hg38). HiCExplorer (v3.6) (28) was then used to create, normalize, and balance the Hi-C interaction matrix. It was also used to analyze and visualize Hi-C data. Briefly, Hi-C matrix was created by ‘hicBuildMatrix -bs 100000 –minDistance 300 –minMappingQuality=15’, normalized by ‘hicNormalize –normalize smallest’, and balanced by ‘hicCorrectMatrix –correctionMethod ICE’. Then the Hi-C matrix was visualized by ‘hicPlotMatrix’. Hi-C matrices of HepG2 and HepG2-215 were compared by ‘hicCompareMatrices’. TADs were identified by ‘hicFindTADs –delta 0.01’ and visualized by ‘hicPlotTADs’. A/B compartments were determined by ‘hicPCA –numberOfEigenvectors 2’ on 100kb binned matrices based on Principal component analysis (PCA). The bwtool (https://github.com/CRG-Barcelona/bwtool) were used to summarize bigWig files, including A/B compartment profiles. Metascape (29) was used to perform functional enrichment analysis between HepG2 and HepG2-215. Additionally, large SVs were identified by applying hic_breakfinder (30) to Hi-C sequencing read alignments.

### scRNA-seq data preprocessing and cell type annotation

We adopted scTE (31) to 10X FASTQ files (converted from 10X BAM files by CellRanger [https://www.10xgenomics.com]) to calculate expression levels of genes and TEs and Viral-Track (32) to detect HBV virus reads in scRNA-seq data. The genome reference index was built by STAR (https://github.com/alexdobin/STAR) using the combined sequence of hg38 and HBV (NC_003977) genomes. Seurat (33) was used to integrate expression levels of genes and TEs and HBV virus information. For quality control, cells with less than 200 UMIs, more than 4000 UMIs, or with more than 15% UMIs mapped to mitochondrial genes were removed. Genes with expression detected in less than 1% cells were also removed. Subsequently, NormalizeData, FindVariableFeatures, ScaleData, RunPCA, RunUMAP, FindClusters, and FindNeighbours from Seurat were applied to preprocess scRNA-seq data for downstream analysis. SingleR (https://github.com/dviraran/SingleR) was used to annotate cell clusters. Differential analysis of gene or TE expression between groups of single cells was performed by FindMarkers from Seurat with default parameters.

### Data visualization

Results were visualized by R packages ggpubr (v0.4.0) or igraph (v1.2.6), unless otherwise specified for Hi-C and scRNA-seq.

## Results

### HBV caused genome-wide changes of chromatin interactions

**Figure 1:**
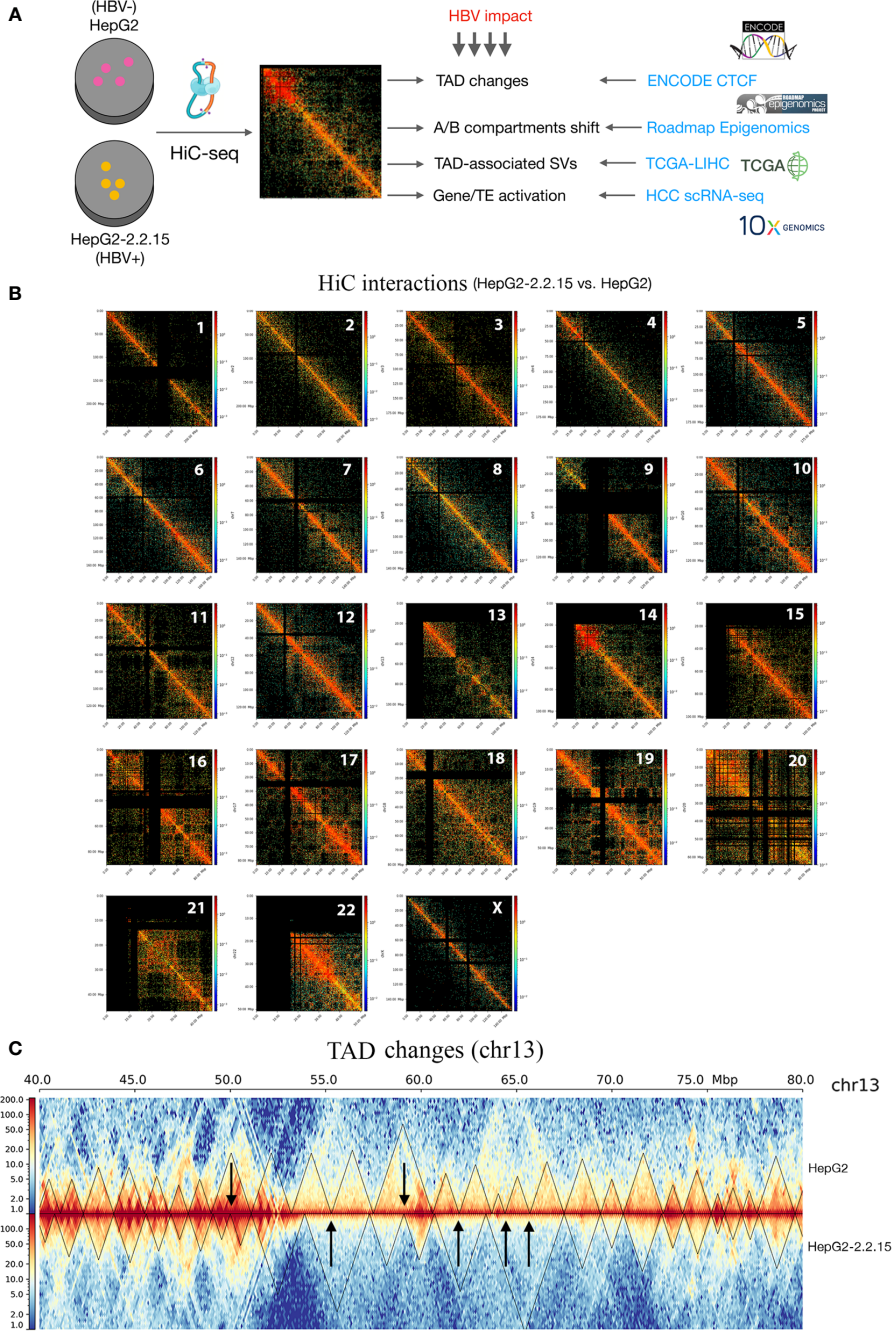
HBV caused global changes of 3D chromosome organizations in liver cancer cells. **(A)** Workflow design of this study, combining our HiC sequencing data with large public datasets to reveal HBV impact on human 3D genome organization in liver cancer patients. TAD, topologically associating domain; TE, transposable element; HCC, hepatocellular carcinoma; SV, structural variation. **(B)** Hi-C interactions comparison between HepG2 and HepG2-2.2.15 by chromosome. Color bars indicate the fold change between the two cell lines. Red indicates more interactions in HepG2-2.2.15 and blue indicates less interactions. **(C)** Chromosome 13 (the 40~80Mbp region) as an example of large-scale TAD changes. Heatmap values were normalized Hi-C interaction frequencies.

To explore the global HBV impact on the human genome spatial organization, we applied the HiC sequencing to two liver cancer cell lines, HepG2 (HBV-) and HepG2-2.2.15 (HBV+) (**Figure 1A**, see Methods). First of all, we observed overall increased frequencies of proximal, indicating more compact chromatins, in HepG2-2.2.15 cells (**Figure S1**), although HBV influenced CIs of individual chromosomes differently (**Figure S2**). We further compared HepG2 and HepG2-2.2.15 CIs by genomic position (1-Mbp bins) for regional differences. Interestingly, we discovered that HBV dramatically increased regional CIs of chromosomes including 10, 13, 14, 17, 19, and 22 (**Figure 1B**). For example, large TAD changes were observed between 50 to 70Mb on chr13, where most smaller TADs were merged into larger TADs and one large TAD was split into two smaller ones (**Figure 1C**). Furthermore, we noticed that five of fourteen (35.7%) reported HBV integration sites in HepG2-2.2.15 cells (34) were nearby TAD boundaries, whose nearby genes included EFNA1, CAMSAP2, GCKR, and SLC4A1AP (**Figure S3**). CAMSAP2 harbored two integration sites and its RNA expression was altered in HepG2-2.2.15 compared to HepG2 (34).

### HBV changed 3D genome topological boundaries favoring cancer metastasis

**Figure 2:**
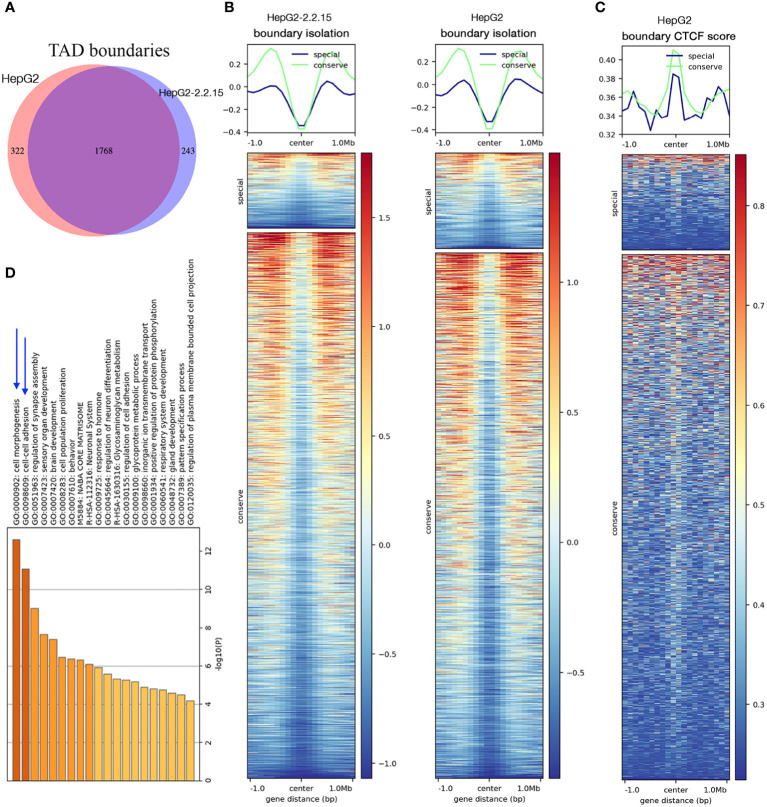
HBV changed topological boundaries that favored cancer metastasis. **(A)** Comparison of TAD boundaries between HepG2 and HepG2-2.2.15. About 10% TAD boundaries were specific to HepG2-2.2.15. **(B, C)** TAD boundaries shared by HepG2 and HepG2-215 showed higher scores (more stable, in green) of TAD isolation **(B)** and CTCF insulation **(C)** than TAD boundaries specific to either cell type (in blue). **(D)** Gene set enrichment analysis of genes within TAD boundaries changed in HBV+ HepG2-2.2.15 cells.

We then compared genome-wide TADs of HepG2-2.2.15 and HepG2. We found a total of 2,333 TADs. About 75.8% (1,768/2,333) TADs were conserved (unchanged by HBV) and 13.8% and 10.4% TADs were specific to HepG2 and HepG2-2.2.15 cells, respectively (**Figure 2A**). To understand the possible reason behind HBV+-specific TADs, we calculated boundary isolation scores and CTCF scores around both types of TADs. We discovered that HBV-altered TADs showed lower isolation (**Figure 2B**) and CTCF scores than conserved TADs between HepG2 and HepG2-2.2.15 (**Figure 2C**), indicating that HBV prefers targeting TAD boundaries less-protected by CTCF and other related proteins. Functional analysis of genes nearby HBV affected TAD boundaries showed that the most enriched pathway was related to cell adhesion (**Figure 2D**), which is closely related to metastasis.

### HBV activated chromosomal compartments with enhancers enriched in inflammatory response

**Figure 3:**
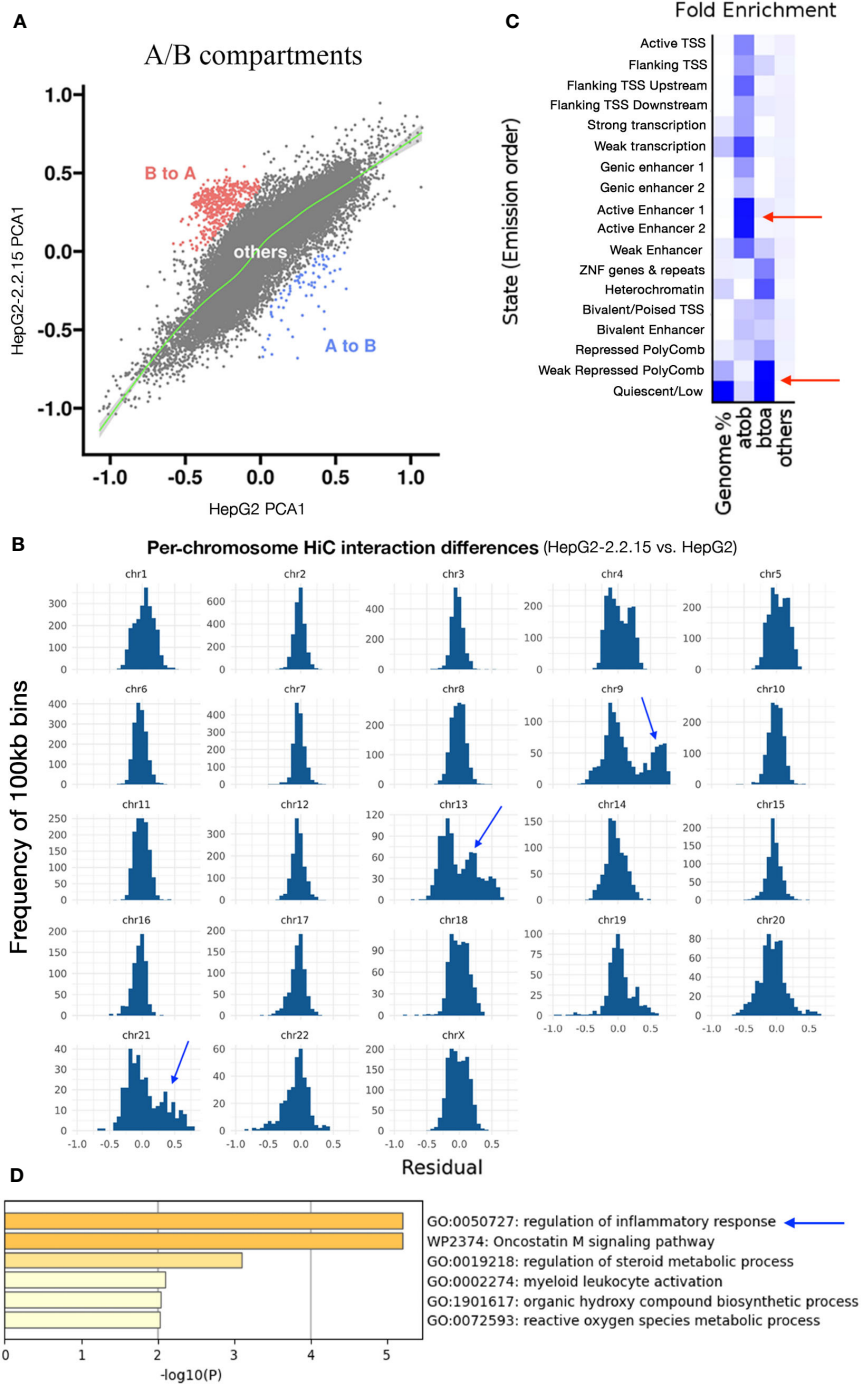
HBV activated compartments related to inflammatory response. **(A)** Comparison of A/B compartmental status of chromatins between HepG2 and HepG2-2.2.15. Each dot represents a 100kb region. **(B)** Distribution of pairwise HiC frequency differences (residues) between HepG2-2.2.15 and HepG2. Y-axis is the number of 100kb bins with corresponding HiC frequency residues shown on the x-axis. **(C)** Chromatin status enrichment of A-to-B and B-to-A compartmental regions. Heatmap values were fold enrichment compared to random background regions; a2b, compartment A to B; b2a, compartment B to A. **(D)** Gene set enrichment analysis of genes targeted by enhancers in A-to-B compartmental regions in HBV+ HepG2-2.2.15 cells.

Next, we calculated compartment A (active or open chromatin) or B (hetero- or closed chromatin) scores (see Methods) for each 100kb regions. We found that more regions changed from B to A than from A to B (**Figure 3A**), implying that HBV resulted in more open chromatins. Dramatic changes of A/B compartments were observed mainly on chromosome 9, 13, and 21 (**Figure 3B**). Chromatin status analysis (35) for these affected A-to-B compartments (repressed) showed that enhancers were mostly affected (**Figure 3C**). We then extracted affected enhancers (n=54) and their associated target genes by querying EnhancerDB (http://lcbb.swjtu.edu.cn/EnhancerDB/) and FANTOM5 (https://fantom.gsc.riken.jp/5/), respectively. Interestingly, we found that these target genes of HBV-repressed enhancers were enriched in the regulation of inflammatory response (**Figure 3D**), which is closely related to cancer. In contrast, B-to-A compartments (active) contained mainly heterochromatin and genomic repeats that were enriched for transposable elements (TE), which have been linked HBV infection (36).

### HBV induced large structural variations potentially disrupting tumor suppressors

**Figure 4:**
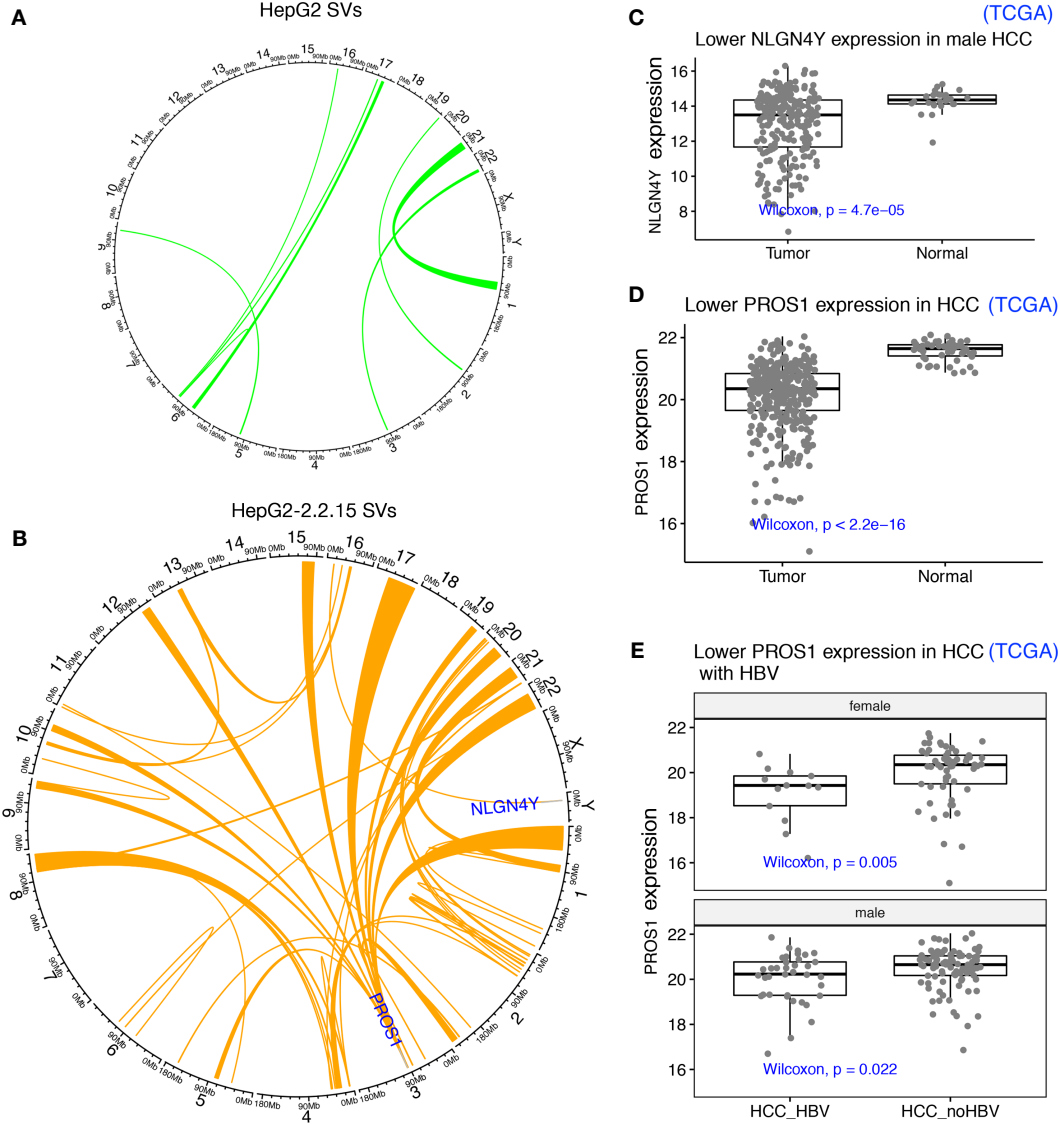
HBV integration caused pervasive large SVs in liver cancer cells. **(A)** SVs identified in HepG2 were shown as links between affected chromosome regions in a Circos plot. **(B)** SVs identified in HepG2-2.2.15. SVs affecting two interesting tumor suppressors (*NLGN4Y* and *PROS1*) in other cancers (not HCC) were highlighted. **(C)** *NLGN4Y* showed lower expression in tumors than in adjacent normal samples from male HCC patients from TCGA. **(D)** *PROS1* showed lower expression in tumors than in adjacent normal samples from HCC patients from TCGA. **(E)** *PROS1* showed lower expression in HBV+ than in HBV- HCC tumors for both males and females from TCGA.

One possible mechanism to induce TAD changes is large structural variations (SVs), which can be detected by using Hi-C data (30). First, we identified SVs using our Hi-C data from both HepG2 and HepG2-2.2.15 samples (**Figures 4A, B**). As expected, since HBV integration induced genome instability and promote oncogenesis in HCC (7), more SVs were found in HepG2-2.2.15 (with HBV integration) than in HepG2. Among these SVs, one affected *NLGN4Y* (neuroligin 4, Y-linked) specifically in HepG2-2.2.15, which is a tumor suppressor in prostate cancer (37) and may contribute to the male dominance of HCC incidence. In male HCC samples from the Cancer Genome Atlas (TCGA), NLGN4Y expression level was lower (Wilcoxon test *P*= 4.7e-5) in tumors than in adjacent normal samples (**Figure 4C**). Interestingly, HBV induced frequent SVs involving many chromosomes at *PROS1* gene (Protein S) in HepG2-2.2.15 (**Figure 4A**), but only one in HepG2 (**Figure 4B**). The immune-related vitamin K-dependent glycoprotein encoded by *PROS1* is synthesized in the liver and is a tumor suppressor that can inhibit metastasis by suppressing the TNF-alpha pathway in lung and breast cancers (38). In TCGA HCC samples, *PROS1* expression levels was significantly reduced in tumors (**Figure 4D**, Wilcoxon test *P*< 2.2e-16). To further support our findings in HepG2-2.2.15 cells, we also observed significantly lower PROS1 in HBV+ than in HBV- HCC samples for both male and female (**Figure 4E**). Of note, no HBV integration was observed in *NLGN4Y* or *PROS1* (5). Further investigate of these two genes in larger patient cohorts is needed.

### Single-cell level analysis revealed that HBV may regulate specific host genes and activate transposons by affecting chromatin interactions

**Figure 5:**
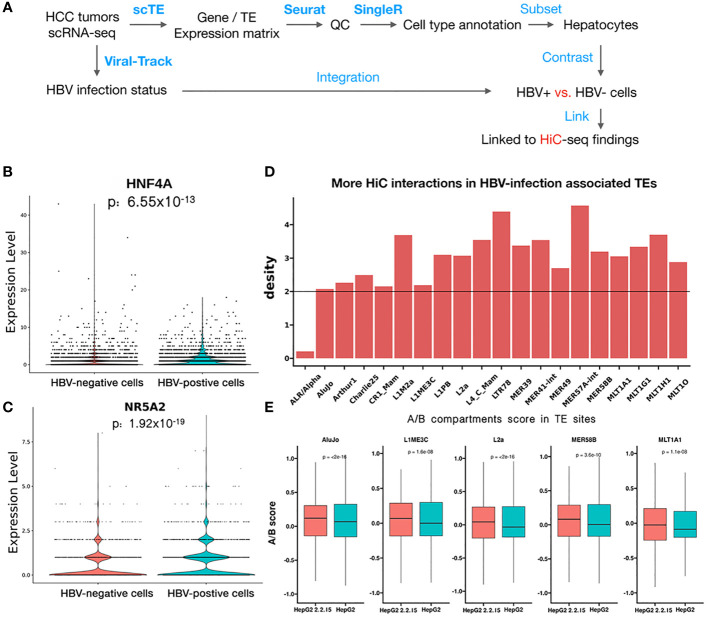
scRNA-seq analysis revealed that HBV infection favorably activated specific genes and transposons. **(A)** Workflow of HCC tumor scRNA-seq analysis. Blue text indicates analysis steps (bold face: name of software tools). **(B, C)** Differential expression of HNF4A **(B)** and NR5A2 **(C)** between individual HBV+ and HBV- hepatocytes. **(D)** More HiC interactions than the genome background (black line) for upregulated TEs identified by scRNA-seq data of HBV+ HCC patients. **(E)** Higher A/B scores (chromatin openness) of five differential TEs in HepG2-2.2.15 than in HepG2 HiC data.

To further support our findings in HiC sequencing, we examined the HBV impact on hepatocyte transcriptomes at the single-cell level using scRNA-seq data from seven HCC tumors (24). After quality control and cell type annotation, we obtained 2,681 hepatocytes, including 1,250 HBV+ hepatocytes, by examining HBV reads in single cells (**Figure 5A**, see Methods). Then, we compared HBV+ and HBV- hepatocytes, resulting in 135 differentially expressed genes and 20 differential TEs. We noticed that two significantly upregulated genes, HNF4A (**Figure 5B**) and NR5A2 (**Figure 5C**), were located in regions with greatly enhanced HiC interactions in HBV+ HepG2-2.2.15 cells. Notably, both HNF4A and NR5A2 have been shown to be able to promote HBV replication (39, 40). For example, the NR5A2-encoded transcription factor, hB1F (hepatitis B virus enhancer II B1 binding factor), can promote HBV replication by regulating the HBV Enhancer II element, which is a critical cis-regulatory element for HBV DNA replication (39). However, we did not find effects of HNF4A or NR5A2 expression on liver cancer patient survival (**Figure S4**), which may be confounded by other oncogenic factors in patients. Moreover, the majority of the 20 differential TEs, such as MER49 and L4_C_Mam, were located in regions with higher frequencies of HiC interactions above genome-wide background TEs (**Figure 5D**). Five of these TEs, AluJo, L1ME3C, L2a, MER58B, and ML1A1, showed higher open compartmental A/B scores in HBV+ HepG2-2.2.15 cells revealed by HiC data (**Figure 5E**). These results indicate that HBV may affect the 3D genome organization of hepatocytes to facilitate viral replication by upregulating specific host factors and TEs.

## Discussion

Here, we performed a comprehensive analysis of HBV impact on 3D genome organization in liver cancer, by combining our HiC sequencing data with large public multi-omics sequencing datasets including scRNA-seq. Collectively, we provide insights into understanding HBV infection and host-virus interactions, which should be important for both liver cancer and other chronic HBV-associated liver diseases that are affecting billions of people worldwide. Based on the HBV-induced genome organization changes related to inflammatory response regulation, our findings may also have implications for novel immunotherapeutic strategies targeting HBV infection.

Of note, during the preparation of this manuscript, another study reported intensive physical interactions between HBV DNA and human chromosomes (9). This study differed from previous studies (8, 9), which mainly focused on active chromatin regions, in several aspects. First of all, we showed that HBV in liver cancer cells, in contrast to short-term HBV infection in primary hepatocytes, induced global chromatin compartmental (A/B) changes, including both active-to-inactive (enriched with inflammatory responses) and inactive-to-active (enriched in repetitive regions, such TEs) chromatin regions. These changes may originate from host DNA damages associated with HBV integration or integrated HBV DNA-formed DNA loop (9) that can potentially function as ecDNA to promote accessible chromatins (22). Second, besides large chromatin compartments, we observed that HBV specifically changed boundaries of vulnerable TADs with low TAD isolation or CTCF insulation scores, which was associated with cancer metastasis. Third, we identified HBV-associated large SVs resulting in 3D genome changes that affect tumor suppressors including *PROS1* and *NLGN4Y*. Finally, we combined public scRNA-seq with our HiC data to further investigate HBV-associated TE reactivation in liver cancer patients, which may indicate better immunotherapeutic responses (41). Collectively, we believe that our study has the potential to better inform future clinical practice related to HBV infection in liver cancer patients.

The limitation of the present study represents our future research direction. Although many of our findings in HCC, the most common primary liver cancer, were consistent with observations from HepG2 and HepG2-2.2.15 cell lines, the cells may not fully represent HCC patients infected by HBV; therefore, more Hi-C sequencing and scRNA-seq of HCC samples and other liver disease samples should be obtained in the future to further evaluate our TAD analysis results in HCC and other liver diseases.

## Data availability statement

The data presented in the study are deposited in the Sequence Read Archive (SRA) repository, accession number PRJNA768726 (https://www.ncbi.nlm.nih.gov/bioproject/PRJNA768726).

## Author contributions

MG and YX conceived the study. MG collected and processed the data, performed the analyses, and interpreted the results with assistance from CJ. MG wrote the manuscript. ZY contributed cell lines and provided scientific input. ZS provided scientific input. LG provided financial support and scientific input. YX and MG provided financial support and reviewed the manuscript. All authors contributed to the article and approved the submitted version.
