# Supplementary material for: Three-dimensional and single-cell sequencing of liver cancer reveals comprehensive host-virus interactions in HBV infection
Source: Front Immunol. 2023 Mar 31;14:1161522. doi: 10.3389/fimmu.2023.1161522 (PMC10102373; doi:10.3389/fimmu.2023.1161522)
Supplement: Supplementary file 1 [file DataSheet_1.docx]

**Supplementary Information**

**Supplementary Figures**


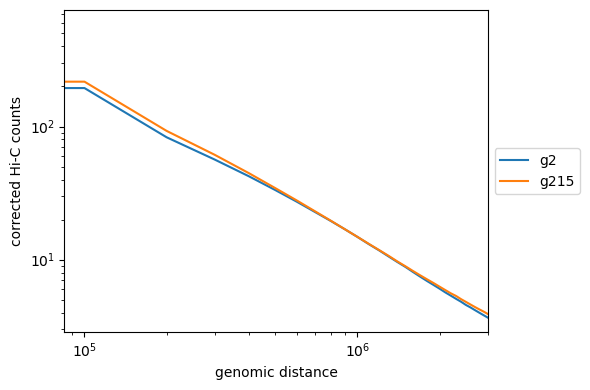


**Fig. S1**. Overall Hi-C interaction frequency at difference genomic distances for HepG2 (g2) and HepG215 (g215).


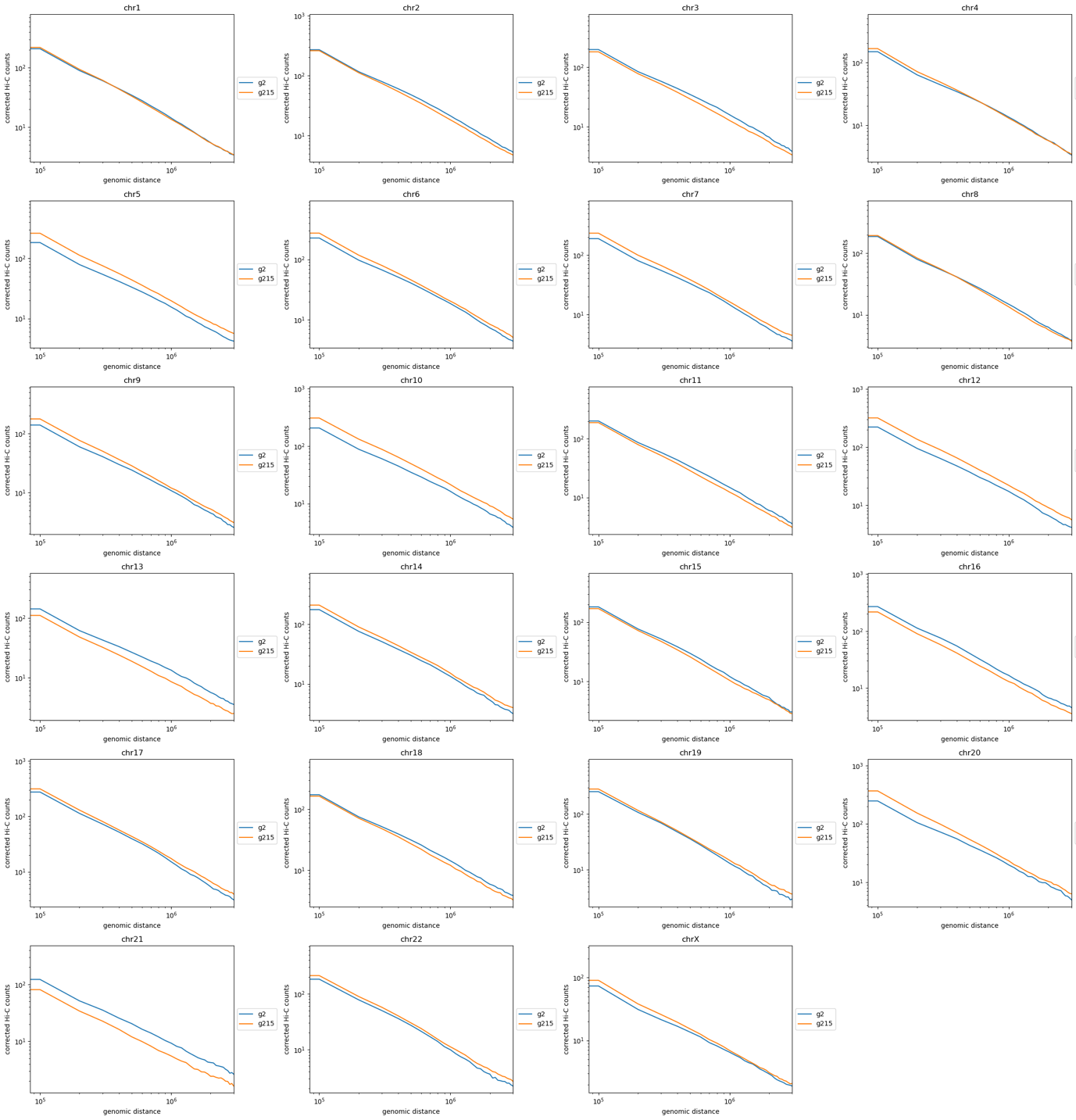


**Fig. S2**. Hi-C interaction frequency at difference genomic distances for HepG2 (g2) and HepG215 (g215) for each chromosome.


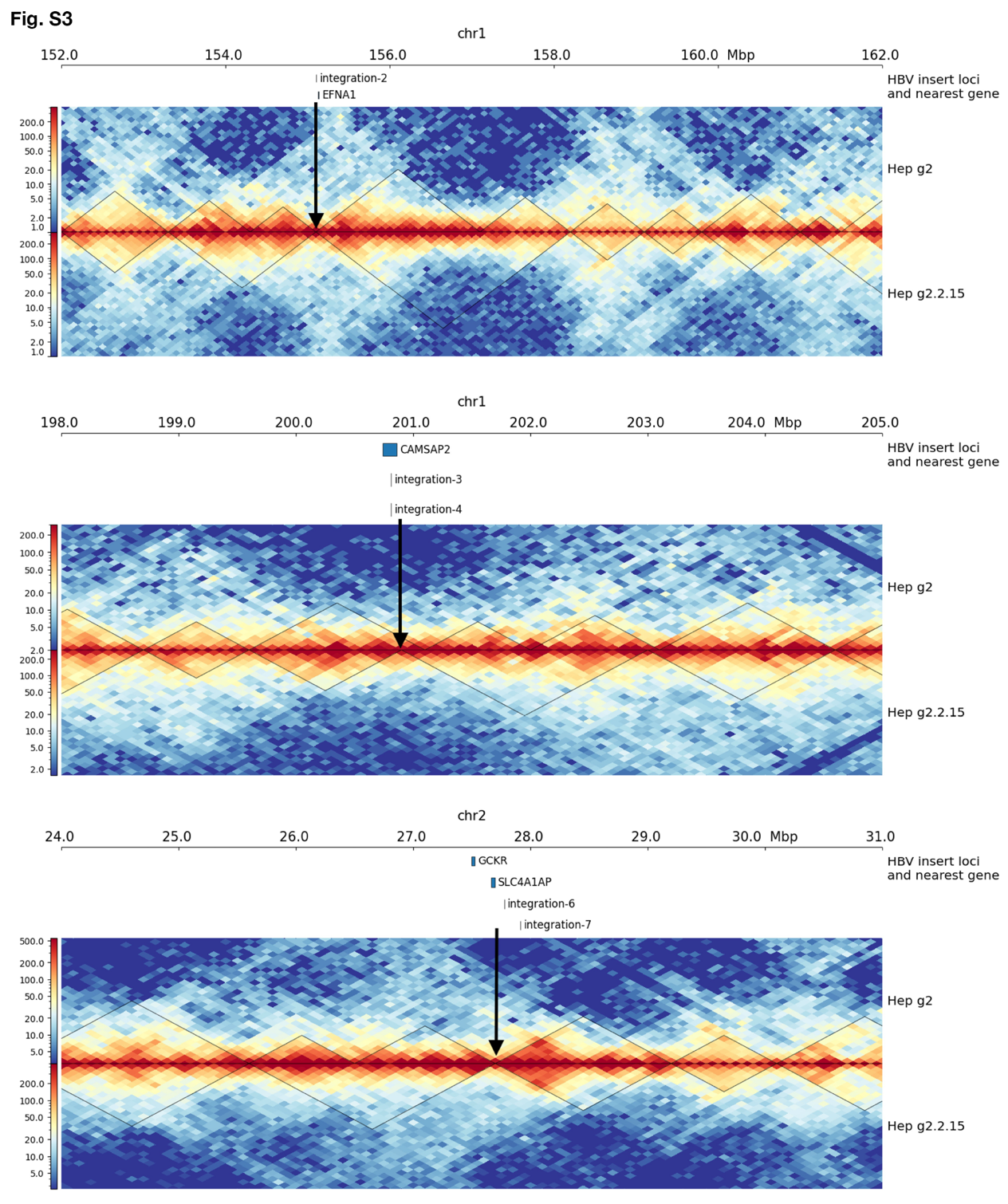


**Fig. S3.** Five of the fourteen reported HBV integration sites (marked by arrows) in HepG215 cells near TAD boundaries.


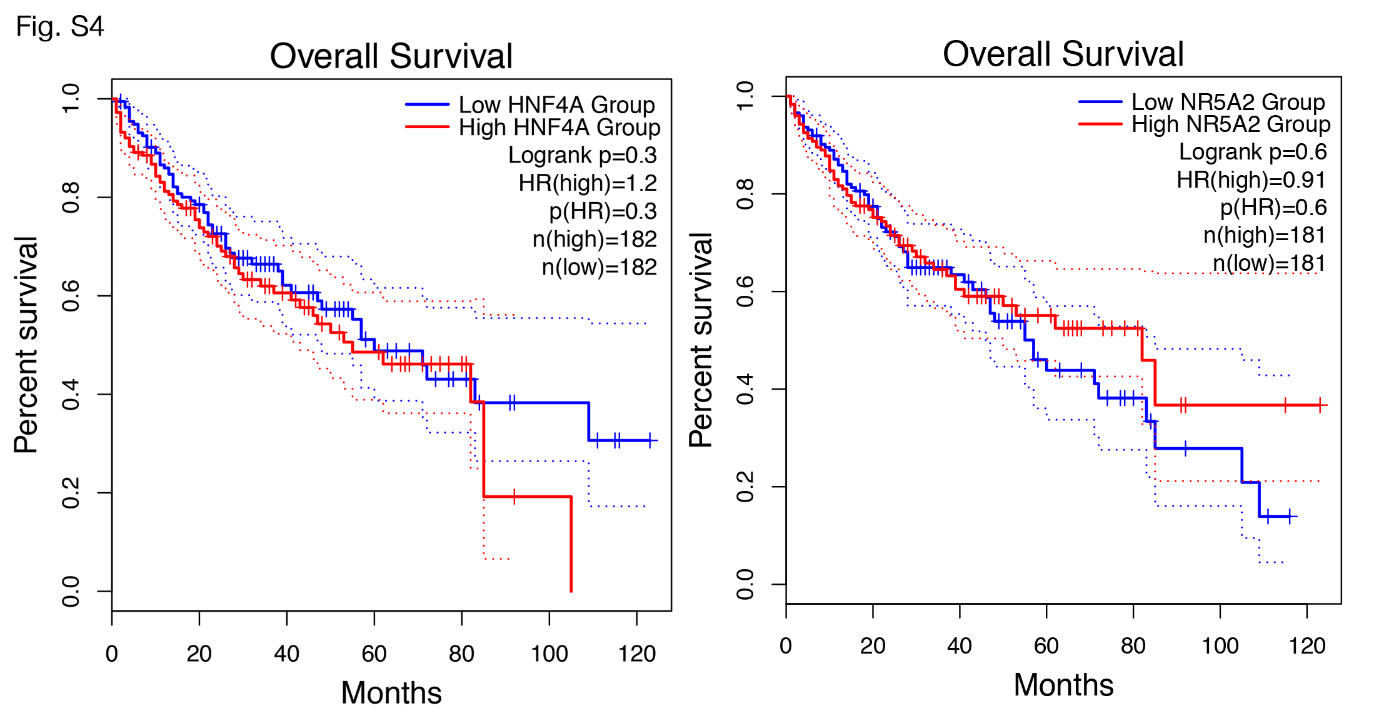


**Fig. S4**. KM curves showing overall survival effect of HNF4A and NR5A2 in TCGA-LIHC liver cancer patients. Figures were created by using the GEPIA2 website (http://gepia2.cancer-pku.cn).
